# Heavy Metal–Gut Microbiota Interactions: Probiotics Modulation and Biosensors Detection

**DOI:** 10.3390/bios15030188

**Published:** 2025-03-14

**Authors:** Liliana Anchidin-Norocel, Oana C. Iatcu, Andrei Lobiuc, Mihai Covasa

**Affiliations:** College of Medicine and Biological Science, Stefan cel Mare University of Suceava, 720229 Suceava, Romania; liliana.norocel@usm.ro (L.A.-N.); andrei.lobiuc@usm.ro (A.L.); mcovasa@usm.ro (M.C.)

**Keywords:** gut bacteria, health monitoring, microbiome, urine analysis, dysbiosis

## Abstract

This study provides a comprehensive analysis of the complex interaction between heavy metals (HMs) and the gut microbiota, adopting a bidirectional approach that explores both the influence of HMs on the gut microbiota populations and the potential role of probiotics in modulating these changes. By examining these interconnected aspects, the study aims to offer a deeper understanding of how HMs disrupt microbial balance and how probiotic interventions may mitigate or reverse these effects, promoting detoxification processes and overall gut health. In addition, the review highlights innovative tools, such as biosensors, for the rapid, precise, and non-invasive detection of HMs in urine. These advanced technologies enable the real-time monitoring of the effectiveness of probiotic-based interventions, offering critical insights into their role in promoting the elimination of HMs from the body and improving detoxification.

## 1. Introduction

Epidemiological evidence suggests that heavy metals (HMs) may play a role in the progression of various metabolic disorders [1], with HM-induced disruptions to the gut microbiota being a contributing factor in their onset and development [2]. Notably, the gut microbiota serves as the first line of defense against the toxic effects of HMs, and there is a dynamic, bidirectional interaction between the two [3]. On one hand, HMs can cause significant alterations in the structure, abundance, and diversity of intestinal microbial communities, while simultaneously influencing their metabolic profiles [4]. On the other hand, the gut microbiota plays a crucial role in modulating the bioavailability of HMs [5], impacting their absorption and metabolism through mechanisms such as altering intestinal pH, redox potential, and the expression of the genes involved in detoxification processes [6]. Through bioaccumulation, binding, and enzymatic transformation, the microbiota can facilitate the excretion of HMs, offering protective effects for the host [7,8]. Furthermore, exposure to HMs, particularly toxic ones, can disturb the gut microbiota, potentially impairing metabolic and physiological functions [9]. Such disruption may contribute to the onset or progression of a wide range of conditions, including cardiovascular diseases, neurodegenerative disorders, ulcerative colitis, cirrhosis, allergies, diabetes, autism, and other inflammatory diseases [10,11].

Recent research has significantly expanded our understanding of the relationship between HMs and the gut microbiota utilizing various models beyond rodents, such as chickens, fish, crayfish, and *Bufo gargarizans*.

The intricate interactions between the gut microbiota and HMs have been increasingly recognized. For instance, cadmium (Cd)-induced dysbiosis in the gut microbiota has been shown to exacerbate liver injury by increasing intestinal permeability in murine models [1]. Studies have consistently reported that HM exposure leads to significant shifts in microbial composition, with a decrease in the abundance of Proteobacteria and Firmicutes and an increase in Bacteroidetes at the phylum level [12].

Probiotics, defined as the mono- or mixed cultures of viable microorganisms that promote health by improving intestinal microbial balance, have shown promise in mitigating HM-induced dysbiosis. Common probiotic species include *Bifidobacterium*, *Lactobacillus*, *Streptococcus*, *Enterococcus*, *Clostridium*, *Bacillus*, and *Escherichia coli*. Among these, certain strains, particularly those from *Lactobacillus*, *Bacillus*, *Bifidobacterium*, and *Clostridium* species, have demonstrated strong resistance to HMs. This resistance is achieved by altering physiological conditions or expressing HM-binding peptides/proteins or detoxification enzymes involved in HM biotransformation. These processes not only reverse HM-induced dysbiosis but also provide protection against HM toxicity [12]. The impact of probiotics on HM elimination can be validated using advanced detection methods for HMs in urine. Various technologies have been developed to detect the concentration and toxicity of heavy metals, including electrothermal atomic absorption spectrometry [13], inductively coupled plasma-optical emission spectrometry [14], and high-pressure liquid chromatography inductively coupled plasma-mass spectrometry, among others [15]. While high-precision methods are valuable, biosensors, point-of-care devices, and similar technologies stand out for their cost-effectiveness, versatility, and robustness, offering rapid, non-invasive, and user-friendly solutions that do not require specialized personnel [16,17,18]. Biosensors operate by detecting signals generated from interactions between analytes and biological components, providing a response proportional to the heavy metal concentration [19]. As defined by the International Union of Pure and Applied Chemistry (IUPAC), biosensors are self-integrated devices that offer quantitative or semi-quantitative data through direct interaction with transducer elements [13]. These methods not only facilitate the assessment of probiotic interventions and HMs status but also provide insights into other health indicators. For example, the presence of copper (Cu) in urine may be associated with Wilson’s disease [20] or urinary tract infections, which have been shown to exert inhibitory effects on certain bacteria [21].

This study offers a comprehensive analysis of the bidirectional interaction between HMs and the gut microbiota. It highlights both the adverse effects of HMs on microbial communities and the potential for probiotics to modulate gut microbiota and elimination of HMs. Unlike previous studies that address these issues separately, our review integrates these findings to provide a deeper understanding of the mechanisms underlying microbial disruption and probiotic-mediated detoxification. In addition, a key innovative aspect of this study is the inclusion of advanced biosensor technologies for rapid, accurate, and non-invasive detection of HM in urine. These cutting-edge tools not only allow real-time monitoring of probiotic interventions, but also improve diagnostic capabilities for HM-related health conditions. By combining knowledge of microbial modulation with state-of-the-art detection methods, this review aims to address critical gaps and provide new perspectives on mitigating HM toxicity and improving gut health.

## 2. Effects of Heavy Metal Exposure on the Gut Microbiota

Exposure to HMs has been linked to significant health risks in both humans and animals particularly through its impact on the gut microbiota [22]. Approximately 60% of the ingested HMs are absorbed in the intestine, where they can cause oxidative stress and damage to the intestinal barrier, which subsequently can lead to increased intestinal inflammation [23,24,25]. While all the studied metals cause microbiota dysbiosis, the specific changes in microbial composition and functional consequences vary between them.

### 2.1. Arsenic

Chronic arsenic (As) exposure has been associated with a range of gastrointestinal disorders including dyspepsia, gastroenteritis, and chronic diarrhea, as well as compromised gut barrier integrity [26,27,28]. Subchronic exposure to As results in significant changes to colonic epithelial structure and impairs intestinal barrier function, primarily through damage to the intestinal microvilli [29]. This disruption of the intestinal barrier is accompanied by an increased production of inflammatory cytokines, such as IL-6, IL-8, and TNF-α, and the generation of oxidative stress in colonic epithelial cells [30,31].

As exposure has been shown to correlate with an increase in pathogenic bacterial populations and a concomitant decrease in commensal bacteria, highlighting its significant impact on gut microbial balance [32,33].

Experimental studies in As-exposed mice showed a marked reduction in the alpha diversity of the gut microbiota, along with a shift in microbiota composition including an increase in Bacteroidetes and a decrease in Firmicutes [34]. For example, a study on 8-week-old mice, exposed to As in drinking water at a concentration of 0.5 ppm and 5 ppm As, found that As exposure induced intestinal inflammation and a decrease in microbial diversity, particularly in the 5 ppm group. Compared to controls, the 5 ppm As exposure decreased the relative abundance of Bacteroidetes and Firmicutes, while Verrucomicrobia significantly increased. Additionally, there was a reduction in the abundance of *Muribaculaceae*, *Alistipes*, *Lachnospiraceae*, *Prevotellaceae*, *Lactobacillus*, and *Alloprevotella*, while the abundance of *Akkermansia*, *Bacteroides*, *Bacteroidales*, *Muribaculum*, *Ruminococcaceae*, *Parabacteroides*, and *Helicobacter* increased following As exposure during the developmental period [35]. In another study by Li et al. [36], exposure to 50 ppm sodium arsenite in drinking water for two weeks in five-week-old laboratory mice led to a significant decrease in the abundance of Bacteroidetes and Tenericutes. At the genus level, the arsenite-exposed mice showed a notable increase in the abundance of *Acetivibrio*, *Pelotomaculum*, *Anaerovorax*, *Anoxybacillus*, *Alistipes*, *Alkalitalea*, *Chryseobacterium*, *Bosea*, and *Curvibacter* compared to the control mice. Conversely, a significant decrease was observed in the abundance of *Clostridium*, *Syntrophococcus*, *Fusicatenibacter*, *Cellulosilyticum*, *Blautia*, *Oribacterium*, *Fastidiosipila*, *Gemmiger*, *Intestinimonas*, *Butyncicoccus*, *Geosporobacter*, *Anaerovibrio*, *Escherichia Shigella*, *Parasutterella*, and *Anaeroplasma*.

These findings collectively highlight the profound and multifaceted impact of As on the gut microbiota. The As-induced dysbiosis not only disrupts microbial diversity and abundance but also exacerbates intestinal inflammation and oxidative stress, contributing to broader systemic effects.

### 2.2. Cadmium

Exposure to cadmium (Cd) is associated with significant disruptions in intestinal barrier integrity and gut microbiota composition. Cd exposure has been shown to reduce the expression and cause abnormal localization of key cell adhesion proteins, such as ZO-1, ZO-2, junctional adhesion molecule A (JAM-A), occludin, and claudin-1, in intestinal epithelial cells. This disruption leads to increased intestinal permeability, which compromises gut integrity and function [8,37]. Notably, Pb exposure mirrors some of the effects of As and Cd by reducing microbial diversity and promoting pathogenic bacteria, yet its effects on the gut microbiota appear more consistent across different studies. For example, Li et al. [36] demonstrated that administering 50 ppm Cd chloride in drinking water to laboratory mice for two weeks significantly decreased bacterial diversity in the intestinal microbiota, compared to controls with a marked reduction in the abundance of Bacteroidetes and Proteobacteria. At the genus level, Cd exposure led to an increase in the abundance of certain bacteria such as *Anaerosporobacter*, *Acidaminobacter*, *Prevotella*, *Barnesiella*, *Parabacteroides*, and *Alistipes*, while the abundance of bacteria like *Syntrophococcus*, *Clostridium*, *Eisenbergiella*, *Coprococcus*, *Blautia*, *Anaerostipes*, *Hespellia*, *Cellulosilyticum*, *Ruminococcus*, *Intestinimonas*, *Gemmiger*, *Caloramator*, *Alkaliphilus*, *Thermotalea*, *Filifactor*, *Gracilibacter*, *Anaerovorax*, *Peptococcus*, *Kandleria*, *Anaerovibrio*, *Desulfovibrio*, *Klebsiella*, *Parasutterella*, *Brevundimonas*, *Acinetobacter*, *Mucispirillum*, *Anaeroplasma*, *Arthrobacter*, and *Clostridium XIVb*, *III*, *IV*, *XI*, *and XVIII* was significantly reduced.

Yang et al. [38] further investigated the effects of varying doses of Cd exposure in rats. They reported that Cd exposure led to a disruption of the microbiota composition, with significant reductions in the abundance of *Prevotella* and *Lachnoclostridium*, while *Escherichia coli–Shigella* populations increased. A 2023 study [39] supported these findings, showing similar changes in the intestinal microbiota composition, with increased levels of pathogenic bacteria such as *Helicobacter* and *Campylobacter* in a mouse model exposed to 3.6 mg/L oral Cd for 23 weeks. Interestingly, unlike As, which primarily shifts microbial populations toward Bacteroidetes, Cd exposure appears to drive a decline in beneficial microbes across multiple phyla while promoting opportunistic pathogens. For instance, a 2020 study [37] reported a significant reduction in the abundance of *Akkermansia muciniphila*, a commensal bacterium known for its role in maintaining intestinal barrier integrity and regulating metabolic homeostasis. Mice exposed to drinking water containing Cd chloride exhibited decreased *Akkermansia muciniphila* populations, further emphasizing the detrimental effects of Cd on gut health. The disruption of the gut microbiota by Cd highlights the compound’s role in promoting intestinal inflammation, microbial dysbiosis, and loss of gut barrier function. These findings underline the importance of further research into mitigating Cd toxicity and exploring strategies to restore the gut microbial balance following exposure.

### 2.3. Mercury

Exposure to mercury (Hg) has been shown to significantly compromise intestinal barrier integrity by reducing the expression of important intercellular junction proteins, leading to increased intestinal permeability and facilitating the absorption of other toxic metals [9]. As such, Hg exposure significantly downregulates the expression of claudin 1, occludin, ZO-1, and JAM1 in colon epithelial cells. Moreover, Hg exposure increases the volume of intestinal cells and membrane permeability without affecting cell viability, thereby enhancing the uptake of other toxic metals under similar physiological conditions [40]. Studies have shown that mercury chloride (HgCl_2_) administration in mice results in significant shifts in gut microbiota composition. For example, a significant increase in the abundance of *Butyricimonas*, *Dehalobacterium*, *Coprococcus*, *Oscillospira*, and *Bilophila* was shown while the abundance of *Sporosarcina*, *Jeotgailcoccus*, *Staphylococcus*, and *Acinetobacter*, was significantly reduced [41]. Further, exposure to 0.4 μg/mL inorganic mercury (IHg) has been shown to induce a phylum-level increase in Tenericutes and a decrease in Verrucomicrobia, indicating significant microbiota alterations compared to controls. At the genus and species levels, this exposure led to a significant increase in the abundance of intestinal pathogenic bacteria, including *Streptococcus*, *Enterococcus faecalis*, *Peptostreptococcus*, *Staphylococcus aureus*, *Pneumococcus*, *Erysipelas*, *Bacillus anthracis*, *Tetanus*, *Spirochetes*, *Actinomycetes*, and *Tuberculosis*, as well as an increase in sulfate-reducing bacteria compared to controls [42].

Finally, exposure of rats to methylmercury, a more toxic form of Hg (0.4 μg/mL, equivalent to 10 μg/kg body weight), led to significant changes in the microbiota composition. Specifically, there was a decrease in the abundance of *Lactobacillaceae*, *Bacteroidaceae*, *Streptococcaceae*, and *Sutterellaceae*, and an increase in *Desulfovibrionaceae*, *Helicobacteraceae*, *Peptococcaceae*, and *Rhodospirillaceae* [43].

These findings highlight the severe impact of Hg on intestinal homeostasis, with broad implications for metabolic, immune, and inflammatory processes. Given its ability to disrupt gut microbiota composition, Hg exposure may contribute to a heightened risk of gastrointestinal and systemic inflammatory diseases.

### 2.4. Lead

Exposure to lead (Pb) has been shown to significantly decrease the expression of the MUC2 gene and tight junction proteins (ZO-1, claudin 1, and occludin) in intercellular junctions, leading to increased intestinal permeability in both mice exposed to low doses over the long term and those exposed to high doses over a short period [44]. A study examining the effects of exposing mice to 10 ppm lead chloride in drinking water (equivalent to 2 mg/kg body weight/day) for 13 weeks found that after just 4 weeks of exposure, there was a reduction in the abundance of *Ruminococcus*, *Clostridiales*, and *Oscillospira*. By the end of the 13-week exposure period, a significant decrease in the abundance of *Lachnospiraceae*, *Blautia*, *Coprococcus*, and *Ruminococcus* was observed [4]. Similarly, Xia et al. [45] examined the effects of 15 weeks of exposure to 0.1 mg/L Pb in drinking water in laboratory mice. Using quantitative real-time PCR of cecal contents, they showed a reduction in the abundance of the Bacteroidetes and Firmicutes phyla. Further, 16S rRNA gene sequencing showed a decrease in Firmicutes and an increase in Bacteroidetes and Proteobacteria phyla. At the genus level, Pb exposure was linked to an increase in *Parabacteroides* and a decrease in *Dehalobacterium*.

Another study showed that the exposure of mice to 1.83 g/L lead acetate ((CH_3_COO)_2_Pb·3H_2_O) in drinking water led to intestinal dysbiosis, characterized by a decrease in beneficial species such as *Akkermansia muciniphila*, *Faecalibacterium prausnitzii*, and *Oscillibacter ruminantium* [46]. Population-based human studies further support these findings. A cross-sectional study involving 696 participants examined urinary Pb concentrations and their association with gut microbiota composition. The results demonstrated a strong correlation between elevated urinary Pb levels and an increased abundance of *Desulfovibrio*, *Eubacterium*, and *Ruminococcus.* Conversely, there was a notable decline in *Clostridium*, *Coprococcus*, and *Pediococcus* [47]. These findings collectively suggest that Pb exposure disrupts gut microbial homeostasis, favoring the proliferation of pro-inflammatory and pathogenic bacteria while depleting beneficial commensal species. This dysbiosis may play a role in Pb-induced systemic toxicity, contributing to inflammatory and metabolic disturbances.

HMs are known to interfere with signaling pathways affecting a variety of cellular processes, including cell growth, proliferation, survival, metabolism, and apoptosis. Their bioaccumulation can lead to diverse toxic effects on different body tissues and organ systems, primarily through their capacity to disrupt antioxidant defense mechanisms, induce oxidative stress, and substitute essential metals in biological processes, leading to cellular damage and increased risk of diseases such as cancer, neurodegenerative disorders, and cardiovascular complications. Table 1 presents the specific effects of some heavy metals on the human body.

## 3. The Role of Probiotics in Reducing Heavy Metal Toxicity

HMs, once accumulated in the body, can cause various chronic conditions and increase the risk of cancer. Due to their persistent nature in the environment, these metals contaminate the soil and enter the food chain, affecting both human health and ecosystems [54,55]. Although traditional detoxification methods exist, they are often costly and may have undesirable side effects. A promising and less invasive alternative is the use of probiotics, live microorganisms that, when administered in appropriate doses, provide health benefits to the host by aiding in the elimination of metals from the body [56,57].

The As resistance genes of certain bacterial genera, such as *Bacteroides*, *Clostridium*, *Alistipes*, and *Bilophila*, enable them to chemically modify As. Through methylation, these bacteria transform As into a less toxic form, thereby ensuring their survival and simultaneously protecting the host organism from the harmful effects of this element [58,59]. The results from a study on the management of As poisoning using probiotics showed that they ameliorated the negative effects of As on oxidative stress and reproductive dysfunction in female rats. The probiotic mixture, containing 137.14 million cells of each *Lactobacillus acidophilus*, *Lactobacillus rhamnosus*, *Bifidobacterium longum*, and *Bifidobacterium bifidum* and 28.57 million cells of *Saccharomyces boulardii*, led to the restoration of antioxidant activities (superoxide dismutase (SOD), catalase, and NPSH), a reduction in malondialdehyde (MDA) and Cd levels, and improved vitamin B12 and estradiol levels. Additionally, they decreased the expression of inflammatory markers and tissue lesions, providing protection against DNA damage and uterine–ovarian necrosis [60]. Another study investigating the effects of probiotics on As toxicity in Wistar rats showed that As increased MDA, myeloperoxidase (MPO), and inflammatory cytokine levels while reducing serum antioxidant defense parameters. Probiotics containing *Lactobacillus* and *Bifidobacterium lactis* demonstrated a significant protective effect, reducing genotoxicity and improving SOD levels, TNF-α, and interferon-gamma (IFN-γ). These beneficial effects were attributed to the probiotics’ ability to decrease the production of reactive oxygen species (ROS) and reduce intestinal inflammation [61].

The protective effects of probiotics against Cd toxicity have been extensively studied. In one study, Wistar rats exposed to Cd exhibited significant metal accumulation in the liver and kidneys, increased oxidative stress (elevated MDA and 8-hydroxy-2′-deoxyguanosine [8-OHdG]), and altered glutathione (GSH) and metallothionein levels. Treatment with *Lactobacillus-* and *Bifidobacterium*-based probiotics and nano-probiotics significantly reversed these effects, with the nano-formulation showing greater efficacy in reducing GSH and 8-OHdG levels [62]. In another study, Djurasevic et al. [63] demonstrated that the administration of *Lactobacillus rhamnosus Rosell-11*, *Lactobacillus acidophilus Rosell-52*, and *Bifidobacterium longum Rosell-175* significantly increased Cd excretion through feces, reducing its levels in the blood, liver, and kidneys, while improving liver function. Similarly, *Lactobacillus plantarum* CCFM8610, a strain with high Cd-binding capacity and antioxidant properties, demonstrated a significant reduction in Cd accumulation in tissues, attenuation of inflammation, and improved gut barrier integrity Cd [8]. *Pediococcus pentosaceus GS4* was also effective in reducing Cd deposition in the liver and intestines, improving membrane integrity, and mitigating Cd-induced intestinal lesions [64]. A comparative study evaluating 13 probiotic strains for their Cd detoxification potential found that *Streptococcus thermophilus* exhibited the highest resistance to Cd and potent antioxidant properties, significantly reducing Cd levels in the bloodstream [65].

Regarding the effects of probiotics on Hg-induced toxicity, Majlesi et al. [66] investigated the efficacy of *Lactobacillus plantarum* and *Bacillus coagulans* in a rat model. Probiotic administration provided significant protection against Hg toxicity by reducing Hg levels in the liver and kidneys and preventing changes in glutathione peroxidase (GPx) and SOD levels. Probiotics also led to a significant reduction in creatinine, urea, bilirubin, alanine aminotransferase (ALT), and aspartate aminotransferase (AST) levels. In another study, *Lactobacillus brevis 23017* protected the intestinal barrier, reducing weight loss and intestinal lesions caused by Hg. It also modulated tight junction proteins and reduced inflammation and oxidative stress through the mitogen-activated protein kinase (MAPK) and nuclear factor kappa-light-chain-enhancer of activated B cell (NF-κB) pathways [67]. Exposure to Hg chloride caused oxidative stress and renal damage, including tubular necrosis, in laboratory mice. Supplementation with a probiotic product containing *Lactobacillus plantarum*, *Lactobacillus delbrueckii* spp. *Bulgaricus PXN39*, *Lactobacillus acidophilus*, *Lactobacillus rhamnosus*, *Bifidobacterium bifidum*, *Streptococcus salivarius* spp. *Thermophilus*, and *Enterococcus faecium* significantly modulated MDA levels and antioxidant enzyme activities, having a beneficial effect on the histopathological lesions induced by Hg toxicity [68].

**Figure 1:**
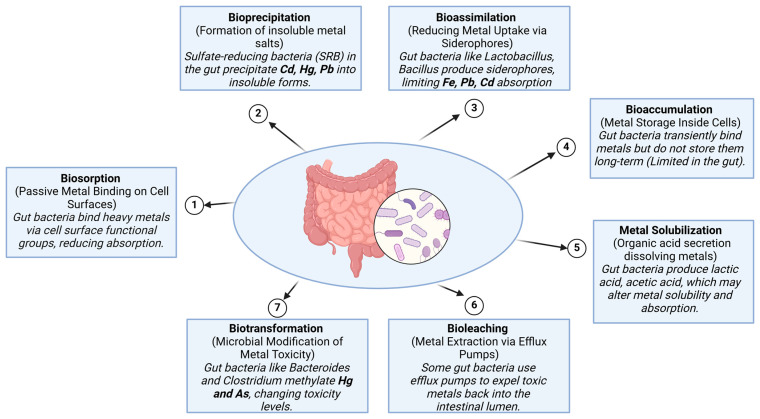
The role of gut microbiota in heavy metal detoxification.

Probiotic-based interventions have also shown promise in mitigating Pb toxicity. Zhai et al. [69] demonstrated that both a supplement containing grape seed extract, tea polyphenols, and *Lactobacillus plantarum CCFM8661*, as well as another supplement including vitamin C, calcium carbonate, zinc acetate, and *L. plantarum CCFM8661*, effectively reduced Pb levels, protecting antioxidant enzyme activities and recovering oxidative stress markers and cognitive deficits in mice. These supplements offered superior protection compared to chelator treatments, and administration during Pb exposure provided a significantly greater protective effect than subsequent administration. Additionally, *Lactobacillus plantarum CCFM8661* protected against Pb toxicity by restoring antioxidant enzyme activity and reducing Pb levels in blood and tissues [70]. The treatment’s efficacy was significantly higher when the probiotic was administered throughout the duration of Pb exposure. Another probiotic studied in Pb toxicity in mice was *Lactobacillus delbrueckii* subsp. *bulgaricus KLDS1.0207*, which was shown to reduce mortality, increase Pb excretion through feces, prevent Pb accumulation in tissues, and improve antioxidant functions in the liver and kidneys [71]. The role of gut microbiota in heavy metal detoxification, highlighting eight key microbial mechanisms is illustrated in Figure 1. Biosorption allows gut bacteria to bind metals on their surface, reducing absorption, while bioprecipitation by sulfate-reducing bacteria (SRB) converts Cd, Hg, and Pb into insoluble salts. Bioassimilation involves siderophore production by *Lactobacillus* and *Bacillus*, limiting Fe, Pb, and Cd uptake, whereas bioaccumulation enables temporary metal storage, though it is limited in the gut. Biotransformation, performed by *Bacteroides* and *Clostridium*, modifies metal toxicity through enzymatic processes such as methylation, reduction, and oxidation [11]. For instance, arsenate reductase reduces arsenate to arsenite, which is more soluble and can be more easily excreted [72], while mercuric reductase converts toxic mercury into elemental mercury, reducing its bioavailability and toxicity [73]. These enzymatic reactions are influenced by various factors such as pH, redox potential, and the availability of cofactors like NADPH and S-adenosylmethionine [74]. Furthermore, bioleaching mechanisms utilize efflux pumps to expel toxic metals back into the intestinal lumen. Lastly, metal solubilization occurs through the bacterial secretion of lactic and acetic acids, which influence metal bioavailability. These mechanisms collectively help regulate metal toxicity and reduce heavy metal absorption in the human body.

**Figure 2:**
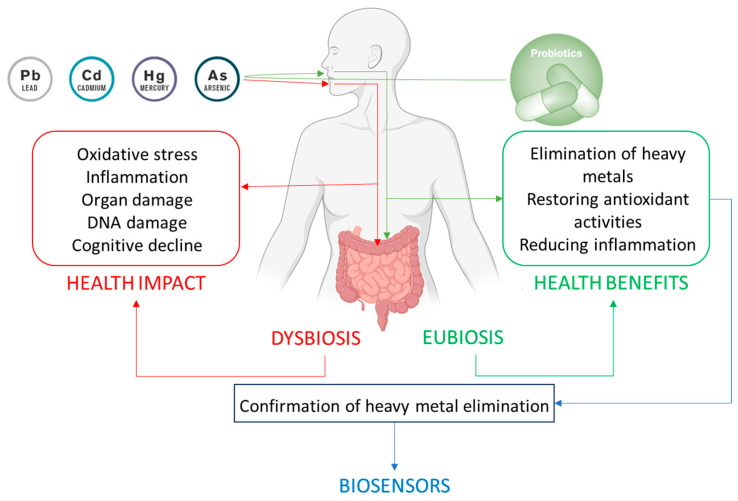
Interaction between heavy metals and gut microbiota, probiotic modulation, and the biosensor-based detection of heavy metals. The figure depicts the dual impact of heavy metal exposure on the body. On one hand, heavy metals exert direct toxic effects on various organs and physiological systems, leading to detrimental health outcomes. On the other hand, they disrupt the balance of the gut microbiota, inducing dysbiosis that exacerbates health issues. Probiotic administration aids in detoxification by reducing metal bioavailability, improving overall health, restoring eubiosis, and enhancing health. Biosensors enable the rapid, accurate, and non-invasive monitoring of heavy metals in urine, providing real-time confirmation of the detoxification process and the efficacy of probiotic-based interventions.

The growing body of evidence underscores the potential of probiotics as a natural and effective strategy for mitigating HM toxicity. Through various mechanisms such as metal binding, biotransformation, competitive exclusion, immune modulation, and gut barrier protection, probiotic bacteria offer a safe and accessible means of detoxification. While further clinical studies are needed to validate these findings in humans, probiotics represent a promising adjunctive therapy for reducing the toxic burden of HMs and minimizing their long-term health consequences (Figure 2).

### Limitations of Probiotic Therapies in HM Detoxification

While probiotic therapies are widely recognized for their health benefits, they also present limitations, particularly in vulnerable populations. Kimse et al. [75] highlight that although generally safe, probiotics can cause mild gastrointestinal symptoms such as bloating, flatulence, or diarrhea, especially during initial supplementation. Rare allergic reactions, manifesting as rashes, itching, or swelling, have also been reported. More concerningly, in immunocompromised individuals, infants, and the elderly, probiotics pose a risk of severe infections, including bacteremia, fungemia, and endocarditis.

Beyond these adverse effects, several other challenges impact the effectiveness of probiotic therapies. One key issue is survivability within the gastrointestinal tract, as probiotics must withstand harsh conditions like acidity and bile salts to be effective [76]. Furthermore, probiotic effects are highly strain-specific, requiring careful selection to ensure therapeutic efficacy. Individual variability in gut microbiota composition complicates standardization, as responses to probiotic treatment can differ significantly between individuals. Regulatory and safety concerns, particularly regarding genetically engineered probiotics, further hinder widespread application, alongside the need for advanced delivery mechanisms to maintain probiotic viability [76,77]. Similarly, other papers [76] highlight not only the challenges in ensuring probiotics survive gastrointestinal conditions, but also their strain-specific nature, and the variability in individual responses due to differences in diet, genetics, and microbiota composition. Variations in manufacturing practices also lead to inconsistencies in probiotic potency and purity, potentially affecting their safety and effectiveness. These challenges underscore the need for continued research and technological advancements to improve the efficacy and reliability of probiotic treatments. Another critical limitation in probiotic prescription practices is the lack of standardized guidelines, leading to inconsistent recommendations among healthcare professionals. Insufficient clinical evidence on the efficacy of many probiotic products raises concerns about their therapeutic benefits. Additionally, variations in regulatory frameworks across different regions contribute to discrepancies in probiotic quality and labeling, complicating prescribing decisions. The vast diversity of probiotic strains makes individualized therapy difficult, often resulting in suboptimal outcomes. Addressing these limitations requires the development of standardized protocols, rigorous clinical research, and improved regulatory frameworks to ensure their safe and effective use.

In the context of probiotics use in mitigating the deleterious effects of heavy metals, several unresolved issues persist. The absence of long-term clinical studies assessing probiotic efficacy and safety remains a concern, while variability in individual responses due to differences in microbiome composition and genetic factors makes treatment outcomes unpredictable [78]. Furthermore, determining optimal dosages and selecting the most effective probiotic strains remains a significant challenge. These issues highlight the necessity for further research to establish strong clinical evidence and standardized therapeutic recommendations. For example, in colorectal cancer therapy that involves monitoring environmental and dietary exposure to heavy metals, given their role in carcinogenesis, probiotics face additional hurdles. Native probiotics often exhibit weak therapeutic efficacy and uncontrolled physiological behavior, limiting their effectiveness against malignancies [79]. Their simple metabolic activities and single therapeutic functions make them less suited for addressing the complexities of cancer treatment. Moreover, the use of viable microorganisms carries risks, including infections, inflammatory responses, and even potential carcinogenic effects. To overcome these obstacles, genetic modifications and synthetic biology approaches have been suggested as means to enhance the therapeutic properties of probiotics, making them safer and more effective for CRC therapy.

## 4. Biosensors for Heavy Metals Detection from Urine

HMs, once accumulated in the body, pose serious health risks contributing to chronic conditions and increasing the risk of cancer [54,55]. These toxic elements are commonly found in bodily fluids such as blood, sweat, and urine, playing a key role in their detoxification and excretion. Thus, monitoring HM levels provides valuable insights into an individual’s toxic exposure and overall health [80]. Given its abundance and ease of collection, urine is an ideal non-invasive diagnostic medium for detecting various human diseases, particularly those affecting the urinary tract. Electrochemical biosensors have emerged as powerful tools for urinary diagnostics due to their exceptional sensitivity, cost-effectiveness, and capability to detect a broad range of target molecules, including nucleic acids and protein biomarkers [81]. These biosensors offer a rapid, portable, and user-friendly alternative to traditional laboratory methods, making them especially valuable in point-of-care (POC) applications. Biosensors are increasingly recognized as highly selective, cost-efficient analytical tools, playing a critical role in early-stage diagnostics and personalized healthcare management. Recent advancements in nanotechnology have significantly improved biosensor performance by enhancing device design, optimizing sensing interfaces, and increasing detection sensitivity. These technological innovations have enabled the development of biosensors tailored for real-time, on-site monitoring, thereby supporting precision medicine approaches [82].

**Figure 3:**
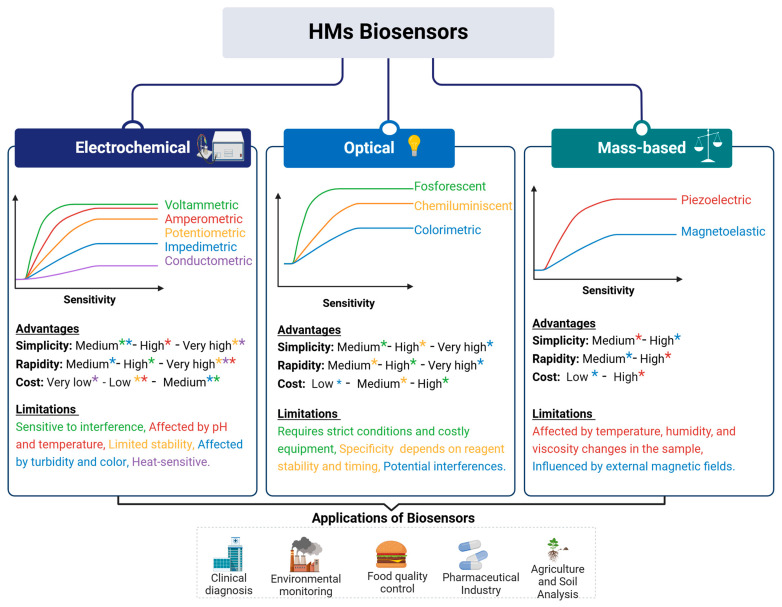
Classification, characteristics, and application of biosensors. Electrochemical biosensors (voltammetric, amperometric, potentiometric, impedimetric, and conductometric) offer high sensitivity and low cost but can be affected by pH, temperature, and sample turbidity. Optical biosensors (fluorescent, chemiluminescent, and colorimetric) provide a lower sensitivity compared with electrochemical biosensors and require strict conditions and costly equipment. Mass-based biosensors (piezoelectric and magnetoelastic) detect metal accumulation based on mass changes, offering high simplicity and rapidity, but can be affected by environmental conditions such as temperature, humidity, and external magnetic fields. At the bottom of the Figure, the key applications of biosensors are highlighted, including clinical diagnosis, environmental monitoring, food quality control, pharmaceuticals, and agriculture. The colors used in the figure correspond to the types of biosensors according to the sensitivity graphs and are later used to highlight the advantages and limitations of each method.

Traditionally, the determination of metal concentrations in body fluids requires sophisticated analytical techniques such as atomic absorption spectrometry (AAS), inductively coupled plasma-mass spectrometry (ICP-MS), and inductively coupled plasma atomic emission spectrometry (ICP-AES) [83,84,85,86]. However, these methods have several limitations, including high operational costs, the need for specialized personnel, and reliance on centralized laboratories. Shipping samples to these labs introduces delays and increases costs, making these techniques impractical for rapid assessment, particularly in low-resource settings and developing countries [87]. Biosensor technology overcomes these limitations by offering a portable, cost-effective, and user-friendly alternative for HM detection. Many metals in urine have a short half-life, reflecting recent exposure and allowing for the real-time monitoring of detoxification interventions, including the effects of probiotic administration [88]. The classification of heavy metal biosensors into three main types: electrochemical, optical, and mass-based biosensors, each with distinct sensitivity, advantages, and limitations, is presented in Figure 3.

**Figure 4:**
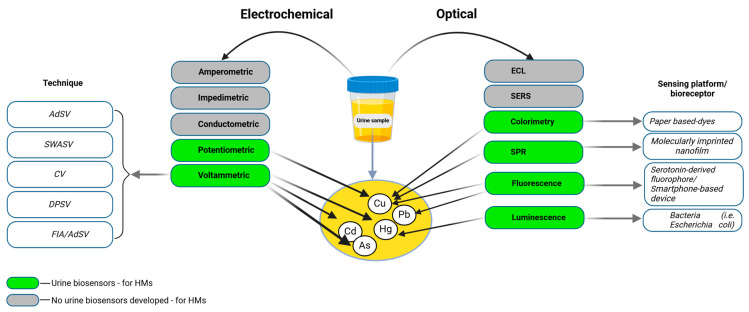
Electrochemical and optical biosensors for urine analysis. Electrochemical techniques, including adsorptive stripping voltammetry (AdSV), square wave anodic stripping voltammetry (SWASV), cyclic voltammetry (CV), differential pulse stripping voltammetry (DPSV), and flow injection analysis combined with adsorptive stripping voltammetry (FIA/AdSV), utilize sensing mechanisms such as amperometric, impedimetric, conductometric, potentiometric, and voltammetric approaches. The green color indicates techniques where urine biosensors for heavy metals are developed, while the gray color signifies the lack of biosensors. Optical techniques include colorimetry, electrochemiluminescence (ECL), Surface-Enhanced Raman Spectroscopy (SERS), surface plasmon resonance (SPR), fluorescence, luminescence, and Förster resonance energy transfer (FRET), which rely on visual or spectral changes for detection. The sensing platforms or bioreceptors associated with these optical methods include paper-based dyes, molecularly imprinted nanofilms, serotonin-derived fluorophores, bacteria (*Escherichia coli*), and smartphone-based devices.

Urine samples for HM determination are preferably collected as first-morning urine using sterile polyethylene or polypropylene containers to minimize the risk of contamination. After collection, the samples are transported to the laboratory in plastic containers and either refrigerated at 4 °C for immediate analysis or aliquoted and frozen at −70 °C for long-term storage. Prior to analysis, the samples undergo acid digestion using concentrated nitric acid (HNO_3_) and hydrogen peroxide (H_2_O_2_) to break down the organic matrix and release HMs. All the equipment used, including laboratory containers, is rigorously cleaned with detergents and distilled water to prevent contamination, ensuring the accuracy and reliability of analytical results [89,90,91]. Figure 4. provides a classification of the techniques and sensing platforms used for detecting HMs (Cu, Hg, Cd, and Pb) in urine samples, categorized into electrochemical and optical methods.

In recent years, a significant number of biosensors have been developed for the detection of HMs in urine, providing innovative, non-invasive solutions for real-time monitoring. A comprehensive summary of these biosensors and their respective methodologies is provided in Table 2.

### 4.1. Arsenic Detection in Urine Using Advanced Biosensors

As, a highly toxic metal linked to severe health issues such as cancer, cardiovascular diseases, and neurological disorders requires effective detection methods to monitor exposure accurately. Advanced biosensors have emerged as promising tools for As detection due to their high sensitivity, selectivity, and low detection limits [109]. Several studies have explored the detection mechanisms for As ions, highlighting significant advances achieved through the development of biosensors that involve aptamers, nanomaterials, and engineered biological systems. Colorimetric biosensors have emerged as simple, cost-effective, and portable and easy-to-use solutions for on-site As detection, making it particularly suitable for in situ applications. They utilize gold or silver nanoparticles, which aggregate and change color upon interaction with As ions [110]. Recent innovations include a 3D-printed device for As (III) detection, further enhancing the practicality of these biosensors [111]. Additionally, inorganic As species (As^+3^ and As^+5^) also exhibit redox properties, enabling spontaneous reactions with chromogenic reagents for precise colorimetric detection [112].

Electrochemical biosensors are widely used for their precision in As detection. These devices utilize modified electrodes incorporating materials such as graphene oxide and gold nanoparticles, which enhance conductivity and surface interactions. Voltammetric techniques, such as square wave anodic stripping voltammetry (SWASV), enable highly accurate detection by pre-concentrating As on nanotextured electrode surfaces and measuring oxidation currents [113].

Molecular biosensors leveraging DNA aptamers provide another powerful approach to As detection. Merulla et al. [114] explored DNA-based biosensors that employ aptamers to target As ions selectively. These biosensors generate detectable changes in response to As binding, such as variations in optical or electrical properties. Functionalization with nanomaterials, such as gold nanoparticles, further enhances sensitivity and selectivity, making them suitable for both environmental and biological assays [115]. Innovative biosensing approaches include the development of genetically engineered bacteria designed to produce measurable signals in response to As exposure. These biosensors utilize biological pathways to detect As, providing a robust and scalable detection platform. Merulla et al. [114] demonstrated that engineered microbial strains can be programmed to fluoresce or change metabolic activity upon As binding, making them highly adaptable for environmental and medical testing.

Jia et al. [116] detailed the development of an advanced whole-cell biosensor using *Escherichia coli DH5α*. Which integrates a positive feedback amplifier to enhance As ion detection. This biosensor utilizes an As-inducible promoter (Pars), the regulatory gene (*arsR*), and the reporter gene *mCherry* creating a fluorescence-based detection system. A positive feedback loop via LuxR autoregulatory elements significantly amplifies output signals, improving sensitivity by an order of magnitude compared to earlier designs. The application of nanotechnology in As biosensing has led to the development of highly sensitive and selective detection methods. Recent studies have demonstrated the effectiveness of carbon dot-MnO_2_ nanocomposites and fluorescent test papers which exhibit high sensitivity and low detection limits for As detection [117]. Functionalization of carbon-based nanomaterials with recognition elements such as aptamers, antibodies, or chemical ligands enables As-induced changes in electrical conductivity, fluorescence, or optical properties, allowing for versatile and efficient detection.

### 4.2. Mercury Detection in Urine Using Advanced Biosensors

Hg concentrations in blood and urine are widely used as biomarkers to assess Hg exposure. A 24 h urine sample is particularly valuable for evaluating the extent of exposure. According to various studies, urine Hg levels below 10 µg L⁻^1^ and blood Hg levels below 20 µg L⁻^1^ are generally regarded as within the normal range [118,119]. Biomonitoring Hg levels in urine can provide valuable information on environmental exposure to inorganic and elemental Hg. This process is particularly important before and during interventions, such as probiotic administration, aimed at enhancing Hg excretion. Additionally, continued monitoring during probiotic consumption is essential to assess whether there is an increase in urinary Hg levels, indicating enhanced Hg excretion [120]. Nanotechnology-based sensors designed for the detection of mercury ions (Hg^2^⁺) include various nanomaterials, such as silver and gold nanoparticles, silica-based materials, magnetic nanoparticles, quantum dots, carbon dots, and electrochemical sensors. These sensors highlight unique properties and mechanisms that enable highly sensitive and selective Hg detection [121]. These technologies leverage the unique properties of nanomaterials, enabling the highly sensitive and selective detection of Hg, a toxic metal that poses significant risks to human health and the environment. Advanced biosensors further enhance detection capabilities by utilizing molecular recognition elements, nanomaterials, and innovative transduction mechanisms.

Aptamer- and antibody-based biosensors are among the most frequently used for detecting Hg^2^⁺. These systems employ recognition elements such as aptamers or antibodies specific to Hg^2^⁺ and rely on transduction methods like electrochemical impedance spectroscopy (EIS) and fluorescence. The integration of nanomaterials, including quantum dots and graphene, improves electron transfer and amplifies signals, allowing for highly sensitive and selective detection. Hg binding triggers optical or electrical changes, which are then measured to quantify Hg levels [115].

Another innovative approach involves peptide-based fluorescent biosensors. A novel biosensor was presented by Sosnowska et al. [122] that utilizes a seven-amino-acid peptide, FY7, that detects Hg^2^⁺ through a 2:1 complex formed between Hg^2^⁺ ions and the tyrosine residue in the peptide. This interaction causes a decrease in fluorescence emission proportional to the Hg concentration. This method enables rapid, sensitive, and selective detection with a low detection limit, requiring minimal sample volumes and being particularly suited for micro-volume analysis using a glass capillary fluorometer. Rajasekar et al. [123] described photoresponsive biosensors which represent an additional advancement, offering exceptional sensitivity and selectivity by exploiting optical changes induced by Hg binding. For instance, azobenzene-based sensors detect Hg^2^⁺ through UV-Vis or fluorescence shifts caused by photoisomerization, while coumarin- and fluorescein-based systems exhibit fluorescence quenching or colorimetric changes upon Hg interaction, facilitating applications such as live-cell imaging and environmental monitoring. Pyrene sensors rely on fluorescence quenching due to Hg^2^⁺ interaction, while quinoline-based sensors detect Hg by altering fluorescence properties, making them ideal for wearable technologies and Internet of Things (IoT) applications [123]. Gold nanoparticle (AuNP)-based colorimetric biosensors are another tool that provides a simple yet effective mechanism for Hg detection. Functionalized AuNPs aggregate in the presence of Hg^2^⁺, leading to a visible color change from red to blue. This method is rapid, cost-efficient, and particularly well suited for on-site and field applications, making it a valuable tool for practical Hg detection [124].

### 4.3. Cadmium Detection in Urine Using Advanced Biosensors

Cd is a widespread HM pollutant that poses significant threats to food safety and human health even at low concentrations. Traditional detection methods are expensive, labor-intensive, and unsuitable for real-time or on-site analysis [125]. This underscores the need for innovative techniques, such as biosensors, which can provide more precise detection and identification of metal compounds like Cd [126]. Monitoring Cd levels in urine is an important biomarker for assessing an individual’s total body burden and potential toxic effects [99]. Genetically engineered biosensors have been developed by Hu et al. [127] to detect HMs such as Cd and Hg with high specificity and sensitivity. One mechanism utilizes a dual-colored bacterial biosensor incorporating CadR and MerR regulators. CadR responds to Cd and Pb by producing prodeoxyviolacein (PDV), emitting a gray-green color, while MerR converts PDV to deoxyviolacein (DV), producing a purple color in the presence of Hg. This biosensor provides dose-dependent dual signals, where gray-green indicates Cd concentrations from nanomolar to micromolar levels, and purple is specific to Hg detection.

Another biosensing approach employs genetically engineered bacterial cells containing artificial Cad operons derived from the natural Cd resistance system of *Pseudomonas putida*. In these systems, CadR, a Cd-responsive transcriptional regulator, activates a promoter upon Cd^2^⁺ binding, leading to the expression of reporter genes like *mCherry* (red fluorescence), *eGFP* (green fluorescence), or *lacZα* (enzymatic activity). These signals indicate Cd concentration. Some designs include a Cd-binding domain on the bacterial surface, enabling the simultaneous detection and removal of Cd [128]. A dual-sensing bacterial bioreporter system utilizes two Cd-responsive regulators, CadC and CadR, coupled with fluorescent reporters such as eGFP for green fluorescence and mCherry for red fluorescence. Upon exposure to Cd, these regulators activate their respective reporters, producing distinct fluorescence signals. This integration allows the simultaneous detection and quantification of bioavailable Cd with enhanced specificity and reduced interference from other metals [129].

Another bacterial biosensor engineered to detect Cd utilizes the production of a visible blue-purple pigment, violacein. This biosensor leverages the Cd-responsive regulator CadR to trigger violacein biosynthesis in the presence of Cd, producing a color change detectable by the naked eye. The violacein signal is stable and measurable at 578 nm, offering a cost-effective, minimal-equipment method for Cd monitoring [130]. Joe M. et al. [131] developed a biosensing mechanism using genetically engineered Deinococcus radiodurans that produces a red pigment, deinoxanthin, in response to Cd. The promoter of the Cd-sensitive gene DR_0659 is activated in the presence of Cd, controlling the expression of the crtI gene responsible for red pigment synthesis. Upon Cd exposure, the bacteria visibly change color from light yellow to bright red, detectable at Cd concentrations ranging from 50 nM to 1 mM. This system relies on a stable, Cd-specific colorimetric reaction with minimal interference from other metals. Similarly to Hg biosensors, Cd biosensors also use aptamers or whole-cell systems to respond to Cd^2^⁺. Nanomaterials such as gold nanoparticles or graphene oxide enhance these systems by improving conductivity and surface area. Electrochemical methods, including differential pulse voltammetry (DPV), are commonly employed for detection, providing high sensitivity and selectivity [115].

### 4.4. Copper Detection in Urine Using Advanced Biosensors

Cu is an essential trace element that plays a crucial role in various biochemical processes and the regulation of physiological functions across all forms of life. It contributes to key physiological activities such as oxidative phosphorylation, angiogenesis, blood coagulation, and antioxidation. Additionally, as a cofactor for numerous enzymes, Cu is indispensable in processes like erythropoiesis, cellular respiration, cholesterol and glucose metabolism, pigment production, and hormone synthesis [132]; however, a high level of Cu is toxic [21]. In addition to the methods presented in Table 1, Cu in urine can also be determined using flow injection analysis with electrochemical detection at platinum disk microelectrodes [133]. The determination of Cu is also highly important for Wilson’s disease (WND). This condition is characterized by increased urinary Cu excretion, which can sometimes exceed 100 µg/day, compared to the normal upper limit of 50 µg/day. In untreated WND patients, urinary Cu excretion (UCE), a reflection of serum non-ceruloplasmin-bound Cu (NCC), is elevated. Consequently, measuring Cu levels in a 24 h urine collection is a common diagnostic test for WND. Urinary Cu excretion exceeding 100 µg/24 h is considered diagnostic for the disease [134].

**Figure 5:**
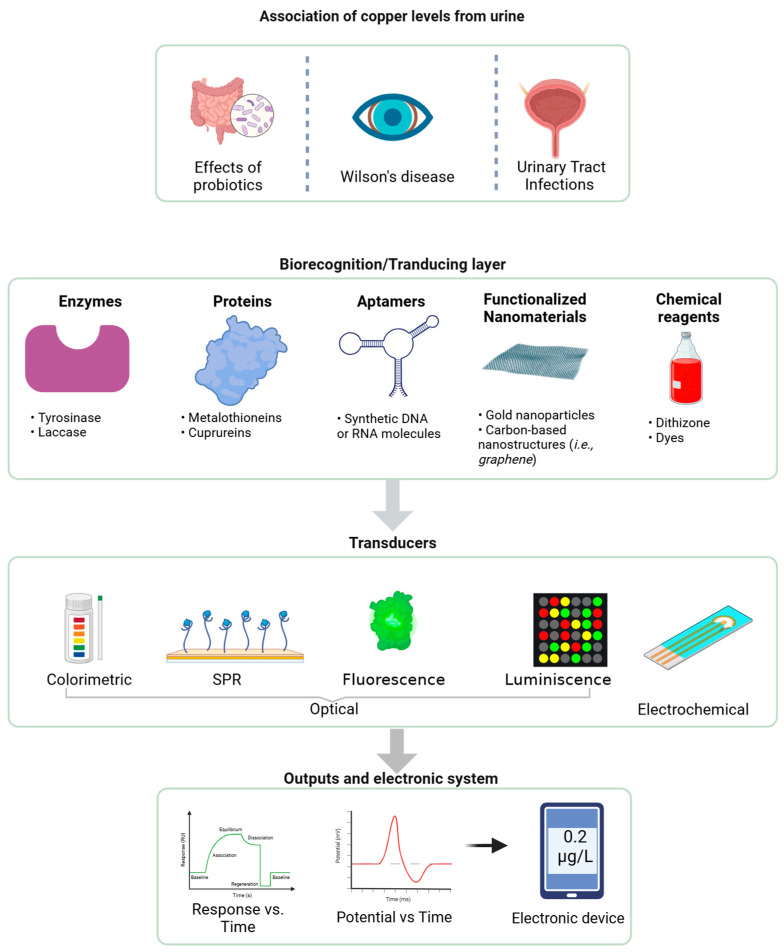
Integrated biosensor system for copper detection in urine (generated with BioRender).

Beyond its metabolic roles, Cu plays a crucial role in defending against urinary tract infections (UTIs) by being mobilized into urine to limit bacterial growth, restricting iron availability through ceruloplasmin, and directly impairing the virulence of uropathogenic *Escherichia coli* (UPEC), highlighting a novel host defense mechanism [135]. Figure 5 shows an integrated biosensor system for detecting Cu levels in urine. It highlights a biorecognition layer incorporating enzymes, proteins, aptamers, functionalized nanomaterials (e.g., graphene), and chemical reagents to ensure selectivity and sensitivity. Transducers such as optical (colorimetric, surface plasmon resonance (SPR), fluorescence, and luminescence) and electrochemical convert interactions into measurable signals. The output is displayed through response–time curves or directly on electronic devices, enabling real-time, accurate, and non-invasive Cu monitoring for diagnostic and therapeutic applications. In addition, it emphasizes its association with probiotics, Wilson’s disease, and urinary tract infections.

### 4.5. Lead Detection in Urine Using Advanced Biosensors

Exposure to Pb in the general population primarily occurs through diet, environmental dust, and contaminated drinking water, with additional contributions from inhaling cigarette smoke and Pb-contaminated air. Blood Pb levels serve as indicators of both recent exposure and the total body burden of Pb. Pb is eliminated from the body through feces and urine, with urinary Pb occasionally used as a biomarker for exposure [91]. Biosensors for Pb detection have emerged as effective tools for environmental and health monitoring. These devices frequently rely on DNAzymes, which catalyze specific reactions in the presence of Pb^2^⁺ ions. Common configurations include electrochemical sensors with modified electrodes, such as those incorporating gold nanoparticles, and optical sensors that detect fluorescence or colorimetric changes. Polyadenine aptamers and split DNAzymes are widely used as recognition elements, as they undergo detectable structural changes upon binding with Pb^2^⁺ ions, allowing for highly selective detection [115]. Other advanced sensors utilize anodic stripping voltammetry techniques incorporating Hg-film electrodes or novel nanostructured materials. Examples include self-assembled monolayers on mesoporous supports and carbon nanotubes, which enhance sensitivity and detection performance through their high surface area and conductivity [100]. A significant breakthrough in Pb biosensing technology is the development of a portable fluorescence resonance energy transfer (FRET)-based biosensor named pMet-Pb. This device features a genetically encoded FRET-based Pb biosensor, Met-Pb 1.44 M1, integrated into a biochip. Upon exposure to Pb^2^⁺, the biosensor undergoes a conformational change that modifies the FRET signal, enabling the quantification of Pb concentrations [101].

## 5. Conclusions and Future Perspectives

HMs exert a profound influence on gut microbiota composition and function, contributing to dysbiosis and a cascade of adverse health effects. The bidirectional relationship between the gut microbiota and HMs underscores the importance of maintaining microbial homeostasis to mitigate toxicity. Probiotic interventions have emerged as a promising strategy to counteract HM-induced dysbiosis by restoring microbial balance, enhancing detoxification processes, and reinforcing intestinal barrier integrity. These findings highlight the therapeutic potential of probiotics not only in HM detoxification but also in broader applications related to metabolic and inflammatory disorders. Beyond microbiome modulation, the integration of advanced biosensing technologies marks a significant step forward in real-time HM detection and exposure monitoring. Biosensors provide a non-invasive, rapid, and highly sensitive means of detecting HMs in urine, allowing for the continuous monitoring of exposure levels and the effectiveness of detoxification interventions. By leveraging biosensors, healthcare providers and researchers can assess the real-time impact of probiotic and dietary interventions, facilitating precision medicine approaches tailored to individual detoxification needs.

Future research should focus on the development of targeted probiotic therapies specifically designed to modulate the gut microbiota in response to HM exposure. Identifying bacterial strains with enhanced HM-binding capabilities will be crucial for optimizing probiotic formulations. Additionally, expanding research on synbiotics, combinations of probiotics and prebiotics, could further enhance the detoxification potential of microbial interventions by providing substrates that promote the growth of beneficial bacteria involved in metal chelation and excretion.

Another key area for advancement lies in the refinement and miniaturization of biosensor technologies. Integrating graphene-based nanomaterials, molecularly imprinted polymers, and artificial intelligence-driven data analysis into biosensor platforms will improve sensitivity, accuracy, and ease of use. The development of wearable or point-of-care biosensors could revolutionize real-time exposure monitoring, allowing individuals to track HM levels in their urine and adjust detoxification strategies accordingly. A promising direction is the combination of microbiome profiling with biosensor data to develop personalized detoxification approaches. By tailoring probiotic treatments, dietary recommendations, and lifestyle modifications based on an individual’s gut microbiota composition and HM exposure levels, healthcare practitioners can implement precision medicine strategies that optimize detoxification outcomes. Furthermore, expanding clinical research into the long-term health effects of chronic low-dose HM exposure and microbiota-targeted interventions will provide deeper insights into preventive and therapeutic measures. Collaborative efforts between microbiologists, toxicologists, bioengineers, and clinicians will be essential in translating laboratory findings into practical applications for environmental health, public policy, and personalized medicine. Ultimately, the integration of microbiome modulation and biosensor-based monitoring offers a powerful and innovative approach to managing HM exposure. Advancements in these fields hold the potential to significantly reduce HM toxicity-related health risks, improve patient outcomes, and pave the way for next-generation detoxification strategies tailored to individual needs.

## Figures and Tables

**Table 1:** Effects of HMs on human body.

Heavy Metal	Effects	References
Arsenic	Heart disease (atherosclerosis, coronary heart disease, peripheral arterial disease myocardial infarction, endothelial dysfunction, thrombosis, hypertension, and stroke) Liver fibrosis Pulmonary disease Muscle cramps and pain Hematopoietic system	[48,49]
Cadmium	Bone damage Lung cancer Renal failure Pneumonitis Proteinuria Heart disease (atherosclerosis, ischemic heart disease, coronary heart disease, dilated cardiomyopathy, heart failure, hypertension, and stroke)	[48,50]
Lead	Impaired voluntary muscle function Heart disease (atherosclerosis, coronary heart disease, peripheral arterial disease endothelial dysfunction, heart failure, thrombosis, hypertension, and stroke) Kidney damage	[48,51]
Mercury	Brain damage Neurodegenerative disorders, gastrointestinal system damage, and kidney damage Respiratory failure Developing fetus damage Reproductive system damage Heart disease (atherosclerosis, ischemic heart disease, myocardial infarction, endothelial dysfunction, thrombosis, hypertension, and stroke) Cancer (lung, skin, colorectal, and brain)	[48,52,53]

**Table 2:** Biosensors for heavy metals in urine.

Technique Used	Metal Ions	Electrode Substrate/Sensing Platform	Linear Range	Limit of Detection (LOD)	Reference
DNA biosensor	Hg(II)	Screen-printed gold electrodes (SPGEs)	10 pM–1 mM	0.11 pM	[92]
Voltammetry	Hg(II)	Screen-printed electrodes modified with gold nanoparticles	-	2.49–7.48 nM	[93]
ASV	Hg(II)	Thin-film gold electrode	99.71–398.82 nM	74.78 nM	[87]
Luminescence	Hg(II)	*Escherichia coli*	0.167 pM–167 nM	0.167 pM	[94]
Whole-cell biosensors (optic)	Hg(II)	Deoxyviolacein M-V (pPmer-vioABCDE), M-DV (pPmer-vioABCE)	1.57–100 nM	0.687 nM for M-V and 0.024 nM for M-DV	[95]
ASV	Pb(II)	Glassy carbon electrodes modified with bimetallic nanoparticle deposits of Ag–Hg and Ag–Bi	-	0.00319 µMand 0.00116 µM	[96]
Whole-cell biosensor	Cd(II)	CadR10 and deoxyviolacein pigment	1.53 nM–100 μM	3.05 nM	[97]
SWASV	Cd(II)	Carbon nanotube film on glassy carbon (CNT-GC)	-	1.9 nM	[98]
CV	Cd(II)	Ag/AgCl reference and platinum wire counter electrode	-	1.9 nM in simulated urine, 5.85 nM (female) and 324 nM (male)	[98]
CV	Cd(II)	Three-electrode setup with BDD electrodes as the working, 3 mm Pt disk as the counter, and Ag/AgCl as the reference.	-	-	[99]
DPSV	Cd(II)	BDD UME array or a macroelectrode, the counter electrode was a graphite rod, and the reference electrode was a KCl-saturated leakless miniature Ag/AgCl	-	0.0116 nM	[99]
FIA/AdSV	Pb(II)	Hg film on the glassy electrode	0–0.241 µM	-	[100]
FRET	Pb(II)	3D-printed frame with 405 nm laser, biochip holder, and smartphone-compatible lens		0.0229 µM	[101]
Fluorescence	Cu(II)	Serotonin-derived fluorophore	-	0.928 ± 0.047 µM	[102]
Colorimetric sensor	Cu(II)	Carboxymethyl gum karaya-capped gold nanoparticles	0.01–1 µM	0.01 µM	[103]
SPR	Cu(II)	Molecularly imprinted nanofilm	0.04–5 μM	-	[103]
Potentiometric	Cu(II)	2-N,N-dimethylcarbamimidoyl modified SPE and CPE	-	1 μM	[104]
Low-field NMR	Cu(II)	QMNPs	-	2 μM	[104]
Colorimetric	Cu(II)	Sodium 8-aminoquinoline-5-azobenzene-4′-sulfonate (SPAQ)	1.57–31.47 µM	0.551 µM	[105]
Microfluidic paper-based chromatic (µPAD)	Cu(II), Zn(II)	1-(2-pyridylazo)-2-naphthol (PAN) reagent	-	0.426 µM 35.9 µg L⁻^1^	[106]
Paper-based sensors	Cu(II)	Multi-dye (Cresol red, Thymol blue, Neutral red)	-	35.09 µM	[107]
Sol–gel optical sensor coupled to a multicommutated flow system	Cu(II)	4-(2-pyridylazo)resorcinol (PAR)	0.0787 µM and 1.26 µM	0.0472 µM	[108]

- no data; Anodic stripping voltammetry (ASV), Cyclic voltammetry (CV), Differential pulse voltammetry (DPSV), Square wave anodic stripping voltammetry (SWASV).
